# Artificial Intelligence and Connected Technologies in Chronic Pain Management: A Scoping Review

**DOI:** 10.1155/prm/5521331

**Published:** 2026-10-18

**Authors:** Paul Elhomsy, Benjamin Reliquet, François Sudria

**Affiliations:** ^1^ Pain Management Department L’Hopital Privé de L’Aube Troyes 10000 France; ^2^ Department of Otolaryngology-Head and Neck Surgery Dijon University Hospital Dijon 21000 France; ^3^ Department of Maxillofacial Surgery Dijon University Hospital Dijon 21000 France

**Keywords:** artificial intelligence, chronic pain, connected devices, digital health, machine learning, pain assessment, personalized medicine

## Abstract

**Background:**

Chronic pain affects over 1.5 billion people worldwide, facing persistent challenges in assessment subjectivity, treatment variability, and scalability (i.e., the limited ability to deliver individualized care to large populations within current clinical systems). Artificial intelligence (AI) and connected health technologies have transitioned from experimental tools to clinical reality in pain management.

**Objective:**

This scoping review maps current AI applications, identifies implementation gaps, and demonstrates the need for systematic technology integration in pain medicine.

**Methods:**

We conducted a targeted literature search across PubMed, Scopus, MEDLINE, Web of Science, and Google Scholar. From 487 identified articles, 23 met inclusion criteria for a detailed analysis. A scoping review approach, following the Arksey and O’Malley framework, provided an integrative perspective on these rapidly evolving applications.

**Results:**

AI implementations span four domains: (1) objective pain assessment via multimodal analysis (facial recognition, neurophysiology, and imaging); (2) personalized treatment strategies using machine learning to optimize therapeutic responses; (3) predictive analytics for risk stratification (postoperative pain and opioid misuse); and (4) real‐time monitoring through connected devices and closed‐loop neuromodulation. Notable applications include AI‐guided spinal cord stimulation and wearable‐integrated pain profiling. Despite demonstrable clinical benefits across these domains, integration remains fragmented due to data governance challenges and educational gaps among practitioners.

**Conclusions:**

AI is demonstrably improving chronic pain outcomes, yet integration remains fragmented due to data governance issues and educational gaps. Professional societies must establish specialized AI committees, clinical guidelines, and structured training. As this transformation occurs, pain specialists must lead this evolution rather than adapt reactively to external changes.

## 1. Introduction

Chronic pain imposes a crushing burden on individuals, healthcare systems, and society, affecting an estimated 1.5 billion people worldwide (Global Burden of Disease Study 2019) and representing a leading cause of disability and healthcare expenditure globally. Despite significant therapeutic advances, core challenges endure the following: the subjectivity of pain assessment [1], highly variable treatment responses [2], and the limited scalability of truly personalized care in current clinical systems [3]. As specialist pain management clinicians, we witness daily the inherent limitations of our current tools when confronted with the immense complexity of chronic pain. It is from this frontline perspective that the integration of new technologies, such as artificial intelligence (AI), appears to us not as an option but as a necessity.

These long‐standing obstacles are now being directly addressed by AI and connected health technologies that have transitioned from experimental concepts to operational clinical tools across multiple domains of pain medicine [4]. Today’s reality includes AI algorithms accurately predicting postoperative pain, machine learning models personalizing medication regimens, and closed‐loop connected devices providing real‐time treatment adjustments [4–6]. Critically lacking, however, is the systematic integration of these proven technologies into mainstream pain medicine practice.

This scoping review aims to map the scope of current AI applications, demonstrate their immediate clinical impact, and identify the structural gaps that demand urgent attention from professional societies.

## 2. Methodology

Following the Arksey and O’Malley scoping review framework, this study maps the current landscape of AI integration in chronic pain management, identifies implementation gaps, and stimulates strategic reflection within the practitioner community, adapted to allow integrative synthesis of a rapidly evolving field where clinical applications are emerging faster than traditional systematic review evidence. We conducted a targeted search of major scientific databases, including PubMed, Scopus, MEDLINE, Web of Science, and Google Scholar between January 2020 and September 2024. Key search terms included “pain management,” “pain relief,” “pain control,” “analgesia,” “pain medicine,” “pain treatment,” “artificial intelligence,” “machine learning,” “deep learning,” “natural language processing,” and “digital health.” These terms were combined using Boolean operators as follows: pain‐related terms were combined with each other using OR (e.g., “pain management” OR “pain relief” OR “pain control” OR “analgesia” OR “pain medicine” OR “pain treatment”), and technology‐related terms were similarly grouped using OR (e.g., “artificial intelligence” OR “machine learning” OR “deep learning” OR “natural language processing” OR “digital health”). The two groups were then combined using AND to maximize search sensitivity and minimize literature gaps. Articles were included if they: (1) were published in English, (2) focused on clinical applications of AI in pain management, (3) reported on implemented technologies rather than theoretical frameworks, and (4) involved human subjects. Exclusion criteria included conference abstracts, veterinary applications, purely theoretical discussions without clinical data, and studies focusing exclusively on acute pain management.

This scoping approach was specifically chosen to allow for an integrative analysis of a rapidly evolving field, where clinical applications are emerging faster than traditional randomized controlled trials (RCTs). Unlike systematic reviews that follow rigid protocols (e.g., PRISMA), this scoping methodology enables the synthesis of diverse study designs and emerging technologies that may not yet have sufficient RCT evidence but demonstrate clear clinical utility.

Following our initial search, 487 articles were identified. After eliminating duplicates (*n* = 124), 363 articles were analyzed against the inclusion criteria. One hundred fifty six articles underwent full‐text screening, and ultimately, 23 articles were retained for this scoping review (Table 1). Data extraction focused on type of AI technology, clinical setting, patient population, outcome measures, and implementation status.

**TABLE 1:** Characteristics of the 23 included studies, organized by AI domain.

Ref.	First author, year	AI domain	AI technology	Study design	Key finding
[1]	Nagireddi et al., 2022	(1) Assessment; (3) Personalized Tx	ML, AI algorithms	Narrative review	AI landscape in pain research
[2]	Casarin et al., 2024	(3) Personalized Tx	AI‐driven personalization	Commentary	Future directions in personalized pain care
[3]	Adams et al., 2025	(4) Real‐time Monitoring	AI roadmap, implementation	Expert review	Current status and requirements for AI in pain medicine
[4]	Antel et al., 2025	(3) Personalized Tx; (2) Predictive Analytics	ML algorithms	Scoping review	Moving toward clinical AI use in pain
[7]	Cascella et al., 2024	(1) Assessment; (2) Predictive Analytics; (4) Monitoring	Multimodal AI, LLMs	Narrative review	AI‐driven diagnostics and multimodal models
[8]	Liawrungrueang et al., 2024	(1) Assessment; (3) Personalized Tx	AI‐assisted MRI analysis	Systematic review	AI improves lumbar disc disease diagnosis via MRI
[5]	Stubberud et al., 2024	(1) Assessment; (2) Predictive Analytics; (3) Personalized Tx; (4) Monitoring	AI, wearables, predictive models	Narrative review	AI applications in headache management
[9]	Bonin‐Guillaume et al., 2016	(1) Assessment	Automated pain detection (Algoplus)	Observational cohort	Validated pain scale for dementia/depression patients
[10]	Zhang et al., 2023	(1) Assessment; (3) Personalized Tx	ML, physiological monitoring	Scoping review	AI improves pain assessment and management
[11]	Meier et al., 2024	(1) Assessment; (3) Personalized Tx	ML, patient subgrouping	Narrative review	AI roles in treatment of chronic pain
[12]	Weatherly et al., 2024	(4) Monitoring	Digital health interventions	Scoping review	DHI effective in chronic pain monitoring
[13]	Petrusic et al., 2024	(1) Assessment; (3) Personalized Tx; (4) Monitoring	Next‐gen AI, wearables	Editorial/review	Next‐gen AI influence on headache research
[14]	Slatman et al., 2024	(3) Personalized Tx	AI‐guided physiotherapy, VR	Development study	Personalized VR‐physiotherapy for low back pain
[15]	Abd‐Elsayed et al., 2024	(2) Predictive Analytics	Predictive ML models	Narrative review	AI applications in pain medicine practice
[16]	Khan et al., 2024	(2) Predictive Analytics	AI risk stratification, opioid prediction	Scoping review	AI for chronic pain prediction and stratification
[6]	Cohen, 2023	(2) Predictive Analytics	Predictive AI, sickle cell pain	Perspective	AI potential and risks in headache medicine
[17]	Prunskis et al., 2025	(4) Monitoring	Closed‐loop SCS, AI optimization	Narrative review	AI‐enhanced SCS efficacy for chronic pain
[18]	Patel et al., 2024	(4) Monitoring	AI‐integrated remote monitoring	Narrative review	AI remote monitoring beyond pain clinic
[19]	Mazzolenis et al., 2024	(1) Assessment; (3) Personalized Tx	Ethics, XAI stewardship	Narrative review	Ethical stewardship of AI in pain/headache
[20]	El‐Tallawy et al., 2024	(4) Monitoring	Telemedicine, digital health	Literature review	Digital health innovations in pain management
[21]	Cascella et al., 2025	(1) Assessment; (3) Personalized Tx	Ethical AI, fairness, XAI	Narrative review	Ethical considerations in AI for pain medicine
[22]	Oliveira et al., 2024	(3) Personalized Tx	AI patient education, XAI	Systematic review	AI role in patient education and accuracy
[23]	Seas et al., 2025	(2) Predictive Analytics; (3) Personalized Tx	ML diagnosis and management	Scoping review	ML in low back pain: diagnosis and care

*Note:* Domains: (1) Objective Pain Assessment; (2) Predictive Analytics; (3) Personalized Treatment; (4) Real‐time Monitoring. XAI = Explainable AI.

Abbreviations: DHI, digital health intervention; LLM, large language model; ML, machine learning; SCS, spinal cord stimulation; VR, virtual reality.

Data extraction also considered the practical requirements for technology deployment. While the reviewed studies primarily demonstrate the clinical efficacy of these tools, they implicitly highlight that successful implementation depends on the clinicians’ ability to interact with complex digital interfaces and interpret algorithmic outputs, suggesting that practitioner readiness is a critical factor in the transition from research to practice.

## 3. Results

Current AI applications in chronic pain management span four distinct domains, each demonstrating varying levels of clinical maturity and implementation.

### 3.1. Objective Pain Assessment and Diagnosis

AI‐powered diagnostic tools have moved beyond laboratory settings into clinical practice. Multimodal approaches now quantify pain using image analysis, facial expression recognition, and neurophysiological signals, with demonstrated readiness for clinical integration [1, 7]. Current applications include sophisticated algorithms that analyze medical imaging (MRI, CT) to identify spinal pathologies with accuracy exceeding traditional radiological assessment [8]. Natural language processing systems utilizing large language models (LLMs) extract detailed pain characteristics from clinical notes, improving diagnostic precision and treatment planning [5, 7].

Automatic Pain Assessment (APA) tools now provide objective, quantifiable pain measures through facial expression analysis and physiological signal monitoring, particularly benefiting patients with communication difficulties [9, 10]. Machine learning algorithms analyze self‐reported pain data alongside physiological measures, creating comprehensive assessment frameworks that synthesize evidence from multiple data sources [10]. These systems integrate real‐time data from smartphones and wearables, combining self‐reported pain levels with physiological indicators to create comprehensive pain profiles [5, 7].

### 3.2. Personalized Treatment Strategies

AI is transforming treatment decisions by modeling rich clinical data, including advanced biological markers, to identify distinct patient subgroups for personalized therapy [1, 11]. Current systems construct individualized pain profiles by integrating genetic, clinical, and psychosocial data, enabling precise treatment customization [1]. Machine learning algorithms identify patient subgroups with differential responses to specific interventions, optimizing therapeutic effectiveness across diverse populations [4, 11].

The clinical reality includes AI systems that predict treatment response and guide individualized therapy through robust validation protocols [7]. These applications encompass systematic approaches to treatment planning, regimen tailoring, and image‐guided interventions [13]. Practical implementations include AI algorithms that recommend personalized medication regimens based on patient characteristics and prior outcome data, dynamically adjusting dosages based on the treatment progress [5, 8]. For chronic low back pain, AI algorithms tailor physiotherapy programs using biomechanical measurements and patient‐specific factors [14]. Advanced systems predict headache onset using wearable sensor data, enabling proactive intervention strategies [5].

### 3.3. Predictive Analytics and Risk Stratification

Predictive models for pain diagnosis, prognosis, and risk stratification represent a mature area of AI implementation [15, 16]. Machine learning models now routinely identify patients at high risk for developing acute postoperative pain, enabling proactive management strategies across surgical specialties [5, 7]. These predictive capabilities extend to identifying patients at risk for poor outcomes and opioid misuse, with demonstrated clinical utility in risk stratification [16]. AI tools predict pain progression, treatment response, and risk of therapy failure, supporting evidence‐based clinical decision‐making [15].

Current systems integrate clinical data with intraoperative nociception measurements to significantly improve postoperative pain prediction accuracy [5]. AI algorithms identify biomarkers through multiple pathways: blood biomarkers, neural signatures via MRI, and physiological patterns from wearable sensors [13]. These comprehensive approaches enable sophisticated pain phenotyping and targeted therapeutic interventions, with validated applications in anticipating pain exacerbations in chronic conditions such as sickle cell disease [6].

### 3.4. Real‐Time Monitoring and Treatment Optimization

Real‐time procedural guidance, perioperative pain trajectory prediction, and dynamic treatment optimization represent advanced implementations of AI in pain medicine [3]. A prime example of this innovation is AI‐enabled closed‐loop spinal cord stimulation (SCS) systems. Traditionally, a clinician manually adjusts SCS parameters during dedicated consultations. However, with closed‐loop systems, AI algorithms manage these parameters, allowing the device to adjust stimulation in real‐time based on physiological feedback. This offers improved pain control and device longevity [17], leading to more stable and personalized analgesia. These systems represent the evolution toward personalized neuromodulation with continuous optimization based on patient response. Connected health platforms provide continuous patient monitoring and dynamic treatment adjustments beyond traditional clinical settings [5, 13, 18]. Current applications include smartphone‐based symptom tracking systems, wearable devices monitoring medication adherence, and AI‐powered virtual assistants supporting patient self‐management. These tools analyze diverse parameters—skin temperature, heart rate variability, muscle tension, and environmental factors—to optimize treatment in real time, with demonstrated clinical effectiveness in chronic pain populations.

AI and connected health technologies demonstrate a capacity to transform clinical outcomes by shifting from standardized protocols to integrated, patient‐centered care ecosystems. However, while these tools have reached a phase of operational maturity in areas such as objective assessment and closed‐loop neuromodulation, their current deployment remains characterized by isolated institutional initiatives rather than a global and concerted strategy within the profession.

## 4. Discussion

The conceptual framework presented in Figure 1 illustrates the current heterogeneity of AI deployment. While diagnostic and predictive tools demonstrate high clinical maturity, real‐time monitoring remains in earlier stages. Achieving the future vision of an integrated ecosystem depends on four systemic pillars: unified governance, explainable AI (XAI) standards, professional education reform, and leadership from professional societies.

**FIGURE 1:**
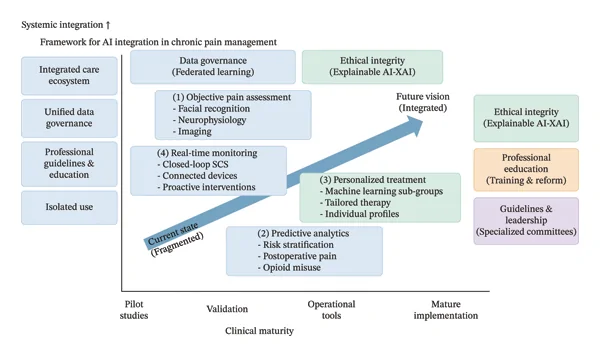
Conceptual framework of AI integration in chronic pain management: current state and future vision.

### 4.1. Synthesis of Current AI Integration in Pain Medicine

This scoping review demonstrates that AI applications in chronic pain management have transitioned from experimental concepts to operational clinical tools across four distinct domains. The evidence reveals a heterogeneous landscape of implementation, with predictive analytics and objective pain assessment showing the highest clinical maturity, while real‐time monitoring technologies remain in earlier deployment phases. Notably, the integration of multimodal AI approaches—combining imaging analysis, physiological monitoring, and patient‐reported outcomes—represents the most promising trajectory for comprehensive pain management optimization.

The clinical impact documented across the 23 reviewed studies indicates measurable improvements in diagnostic accuracy, treatment personalization, and therapeutic efficiency. However, this progress occurs within a fragmented ecosystem where individual institutional initiatives predominate over systematic, profession‐wide integration strategies. This fragmentation constrains the full potential of AI technologies and creates disparities in patient access to advanced pain management capabilities.

### 4.2. Implementation Challenges and Barriers

Data Governance and Privacy Preservation: The primary technical challenge is data governance, stemming from the fragmentation across electronic health records, wearables, and mobile applications, which complicates comprehensive clinical integration [13]. The sensitive nature of pain‐related data—including neuroimaging, genetic markers, and psychosocial records—necessitates robust security protocols [13]. To address this, federated learning emerges as a promising solution for collaborative model development; it allows algorithms to be trained across multiple institutions without ever transferring raw data, thus strictly preserving confidentiality [20].

Ethical Integrity and XAI: Algorithmic fairness is a critical concern, as AI systems trained on biased or unrepresentative datasets risk perpetuating healthcare disparities. This is particularly problematic in pain assessment, where cultural variability and psychosocial factors significantly influence clinical reporting [21]. While AI aims to provide objective clinical measures, these must be interpreted within the patient’s unique psychosocial context to avoid systematic bias. To maintain clinical accountability, the integration of XAI is indispensable. Techniques such as SHapley Additive exPlanations (SHAP) and Local Interpretable Model‐agnostic Explanations (LIME) transform “black‐box” models into transparent systems, ensuring that practitioners understand the specific variables influencing a prediction and maintain ultimate responsibility for the therapeutic act [22].

The Professional Education Gap: The paradox of AI in pain medicine lies in the fact that its greatest strength—the wealth of data from sensors and wearables—is also its primary operational vulnerability due to the lack of unified governance protocols. However, the immediate barrier to systematic adoption remains human rather than technical. Most pain specialists currently lack formal training in data science, algorithmic reasoning, and digital health integration required for effective technology implementation [20]. This educational deficit extends beyond individual practitioners to encompass institutional readiness and the organizational change management capabilities necessary for successful AI integration.

### 4.3. Strategic Actions Required

The evidence presented necessitates urgent, coordinated action across multiple levels of the pain medicine community. Professional societies must immediately establish specialized committees dedicated to AI and digital health integration, with mandates to develop clinical guidelines, establish best practices, and provide continuous technology assessment. These initiatives should include comprehensive mapping of current usage patterns to identify successful implementation models, document barriers, and address specific training deficits across diverse clinical settings.

Medical education reform represents a parallel priority, requiring swift integration of AI literacy and digital health competencies into both undergraduate curricula and continuing medical education programs. Training initiatives must provide practical, hands‐on experience enabling practitioners to evaluate, implement, and integrate AI tools effectively into clinical decision‐making processes.

Patient‐centered research emerges as an essential component of successful AI integration. Understanding how patients currently utilize AI and connected tools for chronic pain self‐management must inform future clinical integration approaches and evidence‐based patient education programs, ensuring that technological advancement serves both providers and patients effectively.

### 4.4. Study Limitations

This scoping review acknowledges several inherent limitations that constrain generalizability and definitive clinical recommendations. The nonsystematic approach, while appropriate for mapping a rapidly evolving field, introduces potential selection bias and precludes quantitative meta‐analysis. The heterogeneity of study designs, outcome measures, and AI technologies across the reviewed literature limits direct comparisons and synthesis of effect sizes. Specifically, a significant majority of the studies identified is observational or cohort designs, with a notable paucity of RCTs, which inherently limits the strength of the evidence. Furthermore, many identified applications are still in the pilot study phase, demonstrating feasibility and potential but not large‐scale, independently validated clinical effectiveness. This predominance of observational studies and pilot implementations reflects the nascent state of AI adoption in pain medicine. While this demonstrates clinical utility and feasibility, it provides limited high‐quality evidence for definitive clinical recommendations. Future systematic reviews utilizing rigorous PRISMA frameworks will be essential to establish robust evidence levels regarding AI tool efficacy and safety in pain management practice.

### 4.5. Future Perspectives: Toward Integrated Care Ecosystems

The trajectory toward integrated AI ecosystems in pain medicine is accelerating. Success will rely on scaling the trust‐based infrastructures (XAI, federated learning) and multimodal data integration discussed previously. Multimodal data combining neuroimaging, molecular biomarkers, and patient‐reported outcomes will enable more sophisticated pain phenotyping and targeted interventions [5, 7, 13].

The ultimate vision encompasses comprehensive AI support throughout the entire patient journey—from early risk detection through real‐time treatment optimization to longitudinal monitoring—integrated seamlessly into electronic health records, telemedicine platforms, and digital therapeutics [5, 14, 23]. Achieving these integrated ecosystems will depend on coordinated efforts across professional education, regulatory frameworks, and technological infrastructure [21, 22]. As the transformation unfolds, the focus must remain on ensuring that these tools serve both clinical efficiency and patient‐centered care.

## 5. Conclusion

In conclusion, this review confirms that AI is no longer a distant promise but an operational clinical reality. Across four principal domains—objective pain assessment, personalized treatment strategies, predictive analytics, and real‐time monitoring—AI tools have demonstrated measurable improvements in diagnostic accuracy, treatment individualization, and therapeutic efficiency. Predictive analytics and objective assessment tools exhibit the greatest current clinical maturity, while real‐time monitoring and closed‐loop neuromodulation remain in earlier deployment phases but show compelling clinical potential. However, this technological readiness is not matched by systematic integration. The primary bottleneck is no longer algorithmic capability, but a set of interconnected human and structural barriers: fragmented data governance, the absence of unified professional guidelines, and a critical educational gap affecting most pain specialists. To bridge this divide, three parallel priorities are urgent: the establishment of AI‐dedicated committees within professional pain societies to develop clinical standards; the reform of medical education curricula to embed AI literacy and digital health competencies; and the development of transparent, explainable AI systems that preserve clinical accountability.

To use a final analogy, we have identified high‐performance Formula 1 engines in these algorithms—but without a sanctioned track, trained drivers, and a coherent race strategy, they cannot safely transform patient care. The pain medicine community must now take the driver’s seat, lead this transformation, and ensure that AI becomes a tool of precision medicine in the service of patients—not an external force to which we simply adapt.

## Author Contributions

Dr. Paul Elhomsy: conceptualization, methodology, writing–original draft, and writing–review and editing.

Dr. Benjamin Reliquet: conceptualization, methodology, and writing–review and editing.

Dr. François Sudria: conceptualization, methodology, and writing–review and editing.

## Funding

The authors have nothing to report.

## Ethics Statement

The authors have nothing to report.

## Consent

The authors have nothing to report.

## Conflicts of Interest

The authors declare no conflicts of interest.

## Data Availability

Data sharing is not applicable—no new data generated, or the article describes entirely theoretical research.
